# Prevalence of musculoskeletal pain among professional drivers: A systematic review

**DOI:** 10.1002/1348-9585.12150

**Published:** 2020-08-18

**Authors:** Leonard Joseph, Miles Standen, Aatit Paungmali, Raija Kuisma, Patraporn Sitilertpisan, Ubon Pirunsan

**Affiliations:** ^1^ School of Health Sciences University of Brighton East Sussex UK; ^2^ Department of Physical Therapy Faculty of Associated Medical Sciences Chiang Mai University Chiang Mai Thailand; ^3^ Karelia University of Applied Sciences Joensuu Finland

**Keywords:** musculoskeletal pain, occupational health, prevalence, professional drivers, rehabilitation

## Abstract

**Objectives:**

Professional drivers are at high risk of developing musculoskeletal pain (MSP) due to risk factors such as prolonged sitting, whole body vibration, awkward posture, and repetitive actions. This review investigates the reported prevalence of MSP among professional drivers.

**Methods:**

An electronic search of Medline (1946 + via OvidSP), Embase (1974 + OvidSP), CINAHL (1982+), AMED, PubMed, and Web of Science from 1990 to July 2019 was performed. Methodological quality of studies was assessed using three quality assessment tools for cross‐sectional, case‐control, and prospective cohort studies. The prevalence of MSP was reported using descriptive analysis.

**Results:**

A total of 56 studies conducted in 23 different countries across a total of 14 types of occupational transport were reviewed. Data of a total pooled population of 18 882 professional drivers were analyzed for MSP. The prevalence of MSP ranged between 43.1% and 93%. The low back was the most frequently reported body region for MSP with a meta‐prevalence rate of 53% (N = 9998). Neck, shoulder, and upper back were the other common regions with high prevalence.

**Conclusion:**

There is a high prevalence of MSP in professional drivers and low back was the most frequently reported body region, followed by neck, upper back, shoulder, knee, hip/thigh, wrist, ankle, and elbow. MSP is complicated in nature and therefore in‐depth exploration of causal relationships between MSP and risk factors is necessary so that appropriate healthcare programs can be initiated to prevent and treat MSP effectively.

## 1 INTRODUCTION

Work‐related musculoskeletal disorders (WRMSDs) are a major public health problem worldwide accounting for between 42% and 58% of all work‐related illness.^1^, ^2^ WRMSDs are defined as impairments of the musculoskeletal system contributed, or aggravated, by work itself or by the environment in which work is performed.^2^, ^3^ Severe WRMSDs can trigger disability, leading to reduced work capability and lost wages.^4^ In addition to work absenteeism, WRMSDs lead to considerable healthcare costs and economic loss to society at all levels.^5^, ^6^ In 2018/2019, the UK Health and Safety Executive (HSE) reported that the prevalence of WRMSDs was 498 000 out of a total of 1 354 000 for all work‐related illnesses, with a 37% prevalence and accounting for 29% of all working days lost due to work‐related ill health.^7^ Recent findings published by the US Bureau of Labor Statistics in 2017 indicated that bus drivers are one of the top three occupations with the highest rates of musculoskeletal disorders, apart from emergency responders and nurses.^8^ Specifically, transit and intercity bus drivers had the highest incidence rates (206 per 10 000 full‐time workers) of musculoskeletal disorders in 2017.^8^ WRMSDs are painful disorders of muscles, bones, nerves, tendons, and other soft tissues, due to workplace activity.^3^, ^9^ This review examined the prevalence of self‐reported MSP as a marker of WRMSDs among professional drivers.

Professional drivers are defined as those people whose key task is to operate a motor vehicle as their main occupational activity.^9^ Previous research has reported high prevalence of MSP in bus drivers (80%),^10^ truck drivers (81%),^11^ and taxi drivers (71%),^1^ with low back pain (LBP) being one of the most commonly reported MSPs.^12^, ^13^, ^14^ Other types of MSP such as shoulder and knee pain are also reported among the professional driving populations.^15^, ^16^ Investigations into the different types of MSP among professional drivers are sparse and the aim of this systematic review is to investigate the current research into the prevalence of different MSPs among professional drivers.

Due to the high prevalence, negative health consequences, and economic impact, MSP is a major occupational health concern for professional drivers. To the researchers’ knowledge, no review has evaluated and reported systematically on the prevalence of MSP among this group of professionals. The current systematic review aims to answer two research questions: (a) What is the estimated prevalence of MSP among professional drivers? (b) What is the prevalence of MSP among drivers who drive light‐to‐moderate and heavy vehicles? The findings of the current review may generate new scientific evidence about the magnitude of MSP among professional drivers. Evidence generated from this may be useful to policy makers, healthcare providers, researchers, and ergonomists to identify occupational risks of professional driving and design appropriate assessment and interventions to reduce rates of MSP among professional drivers. A better understanding of the prevalence of MSP and its risk factors among professional drivers may be beneficial to establish professional guidelines for primary prevention, identify potential work modifications for secondary prevention and provide evidence‐based guidelines to those involved in the decision‐making process of MSP claims associated with professional driving.

## 2 MATERIALS AND METHODS

This systematic review had been conducted and reported according to the guidelines of the Preferred Reporting Items for Systematic Review and Meta‐Analysis Protocols (PRISMA‐P). ^17^

### 2.1 Literature search

An electronic search of Medline (1946 + via OvidSP), Embase (1974 + OvidSP), CINAHL (1982+), AMED, PubMed, and Web of Science was undertaken using a broad strategy. A combination of three main components: professional driving, musculoskeletal disorder, and prevalence/risk were used as MeSH and/or text word search terms. The terms within each component were linked with “OR,” and the three groups were linked with “AND.” A full search strategy based on the Embase literature database is shown in Appendix A. Similar strategies were performed using other databases.

Studies that did not meet the eligibility criteria for this review were excluded through screening titles, abstracts and full texts. Reference lists of included studies were also searched for additional relevant studies. A gray literature search was conducted using the following sources of information: Open Grey, The King's Fund, and WHO (World Health Organization).

### 2.2 Eligibility criteria

The criteria required for inclusion in the review were studies that (a) included professional drivers of >18 years old with at least 1 year of professional driving experience; (b) included professional drivers, defined as those whose main task was to operate a motor vehicle in traffic conditions; (c) had a primary purpose of examining the prevalence of MSP among professional drivers; (d) were published in peer‐reviewed English language journals; (e) utilized cross‐sectional, case‐control, or prospective cohort study designs and reported prevalence of MSP; and (f) reported results on prevalence for MSP along with risk factors associated with professional driving.

Studies that were excluded (a) had no specific population (eg, too broad); (b) reported incidence of MSP without prevalence; and (d) were non‐scientific studies (eg, editorials, commentaries), literature reviews, reporting only treatment of pain, basic sciences, or cadaver studies.

### 2.3 Screening process

Search results were exported into Endnote™ (EndNote x8 for Windows version) to check for duplicate studies which were removed accordingly. Bibliographic records were then exported from Endnote™ into Microsoft Excel to enable further manual deletion of duplications. Initial screening was conducted on the title and abstract of studies by one reviewer and cross‐checked by a second reviewer. The second level screening evaluated full‐text reports for studies deemed potentially eligible after the first screening. Disagreements among reviewers were resolved by discussion and reflection with the third reviewer.

### 2.4 Methodological quality assessment

The methodological quality of studies was assessed independently by two reviewers using three quality assessment tools. A risk of bias tool was used to assess the quality of cross‐sectional studies.^18^ This tool assesses external validity through four items (1‐4), and evaluates internal validity using six items (5‐10). Case‐control and prospective cohort studies were assessed using the Newcastle‐Ottawa Scale for observational studies.^19^ A case‐control version of the Newcastle‐Ottawa Scale was used for case‐control studies consisting of nine items assessing selection, comparability and exposure. The Newcastle‐Ottawa Scale for cohort studies was used for prospective cohort studies that assessed nine items of selection, comparability, and outcome.

The overall methodological quality of each included study was rated as being high quality (low risk of bias), medium quality (high risk of bias), or low quality (very high risk of bias). Total scores from the risk of bias tool and Newcastle‐Ottawa scales were categorized into three groups: Very high risk of bias (0‐4 points), high risk of bias (5‐6 points), and low risk of bias (7+ points).^20^ This method is consistent with Grades of Recommendation, Assessment, Development and Evaluation and Cochrane approaches.^21^

### 2.5 Data extraction

The study characteristics extracted from the reviewed studies included information on the authors, year of publication, country, study design, number of participants, type of vehicle driven, and aim of study. If reported, further information regarding participants' mean age was collected. For information regarding the prevalence of MSP, the type of professional driving (vehicle driven), type/area of MSP, prevalence duration, and results of prevalence were collected. If the case‐control and prospective cohort studies provided a baseline cross‐sectional prevalence rate of MSP among the drivers, the reported prevalence rate was extracted for this review.

### 2.6 Analysis of data

The prevalence of MSP presented among the different studies was reported using descriptive analysis. The meta‐prevalence estimate of MSP for each specific body region reported for professional drivers was calculated by weighing the studies according to their sample size within pooled samples. This was done using the meta‐prevalence estimate formula in Microsoft Excel; thus, giving a meta‐prevalence estimate of MSP for each body region.^22^ Where available, prevalence rates from the median prevalence duration were used for calculation. In this review, the most commonly reported prevalence duration was 12 months. For this reason, if data for 12‐month prevalence were available, they were used to calculate the estimated meta‐prevalence. In addition, subgroup descriptive analysis was conducted to calculate the meta‐prevalence estimate of MSP among professional drivers in two subgroups, namely low‐moderate vehicles and heavy vehicles. For subgroup analysis, buses, trucks, and cranes were classified as heavy vehicles while all other types of vehicles were classified as low‐moderate vehicles.

## 3 RESULTS

### 3.1 Study selection

**FIGURE 1:**
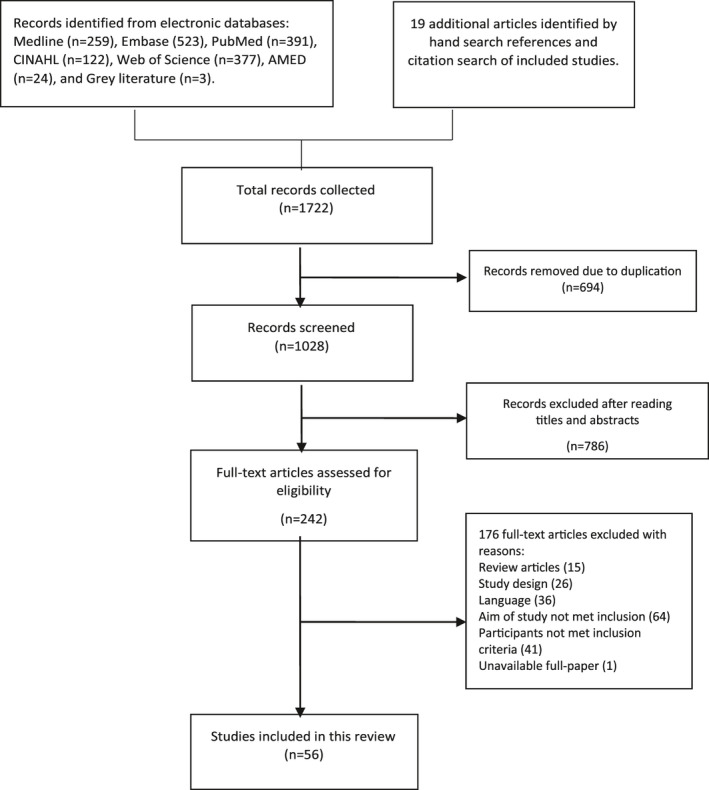
Flowchart of study selection process

The search strategy yielded a total of 1028 citations, 242 of which were deemed potentially relevant at the first cycle of screening. On further review, 56 studies satisfied the eligibility criteria. The PRISMA flowchart explaining the process of selection is shown in Figure 1.

### 3.2 Study characteristics

The 56 studies involved a total of 18 882 participants, included in 48 cross‐sectional studies, five prospective cohort studies, and three case‐control studies. The characteristics of the studies included are summarized in Table 1. The age of participants ranged from 20 to 71 years with a mean age of 42.8 years. Of those studies reporting mean years of driving experience and mean hours driving per day, results showed 13.1 years and 9.6 hours, respectively. The included studies were conducted in 23 different countries, nine in the United Kingdom, eight in the USA, and seven in Italy. Studies from other countries included the Netherlands (four) and India (four), Malaysia (three) and Iran (three), Israel, Ghana, Nigeria, Taiwan, China, Canada, and Japan (two each), and one study each from Brazil, Sweden, Finland, Poland, Norway, South Africa, Germany, Turkey, and Hong Kong. A total of 14 types of occupational transport were represented by these studies, most commonly buses in 18 studies, trucks in 11 studies, and taxis in 10 studies. Other vehicles included transit vehicles, minibuses, tractors, straddle carriers, police cars, rally cars, delivery vans, cars, garbage trucks, earth moving vehicles, cranes, and forklifts.

**TABLE 1:** Characteristics of the included studies

Author (year)	Country	Study design	N	Vehicle	Mean age (y)	Aim of study
Abledu, Offei and Abledu (2014a)^2^	Ghana	Cross‐sectional	148	Minibus	33	Determine prevalence of MSD
Abledu, Offei and Abledu (2014b)^40^	Ghana	Cross‐sectional	210	Taxi	32.1	Determine prevalence and predictors of MSD
Akinpelu et al (2011)^41^	Nigeria	Cross‐sectional	159	Various	40.4	Determine prevalence, distribution, illness perceptions and health seeking behavior
Alperovitch‐Najenson et al (2010a)^15^	Israel	Cross‐sectional	361	Bus	46	Evaluate prevalence and association between risk factors and neck pain
Alperovitch‐Najenson et al (2010b)^42^	Israel	Cross‐sectional	361	Bus	46	Evaluate prevalence and association between risk factors and LBP
Aminian et al (2016)^43^	Iran	Cross‐sectional	734	Truck and Taxi	41	Evaluate prevalence of MSDs and compare between truck and taxi
Anderson (1992)^44^	USA	Case‐control	128	Bus	NR	Examine the extent of spinal problems and compare to non‐ driving control
Andrusaitis, Oliveira and Barros Filho (2006)^45^	Brazil	Cross‐sectional	410	Truck	40.1	Investigate prevalence of LBP and check possible associated risk factors
Anjomshoae, Rani (2013)^46^	Malaysia	Cross‐sectional	131	Bus	48.3	Assess the MSDs and psychosocial risk factors in Malaysian bus drivers
Boshuizen, Bongers, and Hulshof (1990)^47^	Netherlands	Cross‐sectional	577	Tractor	NR	Investigate prevalence of back pain in relation to past exposure of WBV
Boshuizen, Bongers, and Hulshof (1992)^48^	Netherlands	Cross‐sectional	196	Truck	NR	Investigate self‐reported LBP in relation to past exposure of WBV
Bovenzi (2009)^49^	Italy	Prospective cohort	537	Various	40	Investigate relation between measures of daily/cumulative vibration exposure and LBP outcomes
Bovenzi (2010)^50^	Italy	Prospective cohort	202	Various	40	Investigate relation between measures of daily/cumulative vibration exposure and LBP outcomes in LBP free baseline drivers
Bovenzi (2015)^36^	Italy	Prospective cohort	537	Various	40	Investigate the occurrence of neck and shoulder pain in relation to occupational risk factors
Bovenzi and Betta (1994)^51^	Italy	Cross‐sectional	1155	Tractor	43	Investigate the relationship between WBV dose, perceived postural load and low back complaints
Bovenzi et al (2006)^52^	Italy	Cross‐sectional	598	Various	40	Investigate prevalence of LBP and association between LBP, WBV exposure, physical load and psychosocial variables
Bovenzi et al (2015)^53^	Italy	Prospective cohort	537	Various	40	Investigate relation of sciatic pain to measures of WBV exposure and internal spinal load
Bovenzi and Zadini (1992)^23^	Italy	Cross‐sectional	234	Bus	42.5	Investigate the prevalence of several types of back pain in relation to WBV
Burdorf, Naaktgeboren and Degroot (1993)^54^	Netherlands	Cross‐sectional	95	Straddle‐carrier	41	Investigate the prevalence of LBP in three groups of sedentary workers and determine associated risk factors
Burgel and Elshatarat (2017)^55^	USA	Cross‐sectional	129	Taxi	45.3	Identify associations between psychosocial risk factors and LBP in taxi drivers
Chen et al (2004)^16^	Taiwan	Cross‐sectional	1242	Taxi	44.5	Explore the postulated association between daily driving time and knee pain
Chen et al (2005)^56^	Taiwan	Cross‐sectional	1242	Taxi	44.5	Examine LBP in taxi drivers and its association with prolonged driving
Feng (2018)^57^	China	Cross‐sectional	162	Taxi	37.6	Assess the correlations between the severity of musculoskeletal disorders (MSDs) and aberrant driving behaviors
Gangopadhyay and Dev (2012)^58^	India	Cross‐sectional	160	Bus	35.8	Investigate the prevalence of LBP and determine social or professional restrictions
Geete et al (2013)^10^	India	Cross‐sectional	60	Bus	NR	Investigate prevalence of MSK pain and analyse risk factors associated
Greiner and Krause (2006)^59^	USA	Cross‐sectional	66	Transit	47.2	Determine whether risk factors are associated with prevalence of MSDs
Gyi and Porter (1998)^60^	UK	Cross‐sectional	80	Police Car	37.7	Investigate the prevalence of MSK trouble in police car drivers and non‐drivers
Jadhav (2016a)^61^	India	Cross‐sectional	178	Bus	NR	Compare the prevalence of chronic LPB and find association with occupational risk factors
Kaila‐Kangas et al (2011)^62^	Finland	Cross‐sectional	2323	Various	NR	Investigate whether driving exposure is associated with clinically defined sciatica or other low back syndromes
Kim et al (2016)^63^	USA	Cross‐sectional	96	Truck	NR	Characterize WBV exposures in truck driving and determine association between WBV exposures and MSK outcomes
Krause et al (1998)^64^	USA	Prospective cohort	1854	Transit	NR	Investigate psychosocial risk factors as predictors of work‐related spinal injuries, controlling for physical workload
Krause et al (1997)^65^	USA	Cross‐sectional	1449	Transit	42.4	Examine the relation between physical workload, ergonomic factors and prevalence of back and neck pain
Laal et al (2017)^66^	Iran	Cross‐sectional	60	Bus	40	Examine prevalence and severity of MSDs as well as anthropometric dimensions
Lalit, Soni and Garg (2015)^67^	Poland	Cross‐sectional	300	Bus	42.6	Investigate the prevalence and characteristics of WRMDs in city bus drivers
Magnusson et al (1996)^13^	USA	Case‐control	228	Bus and Truck	41	Establish the effect of mechanical and psychosocial factors in reporting back, neck and shoulder pain and work loss
Mansfield and Marshall (2001)^68^	UK	Cross‐sectional	90	Racing car	34	Investigate the prevalence of MSKSs after race
Miyamoto et al (2008)^69^	Japan	Cross‐sectional	1334	Taxi	51.5	Investigate the prevalence of low back symptoms and associated risk factors
Miyamoto et al (2000)^70^	Japan	Cross‐sectional	153	Truck	41	Determine the actual situation of drivers' LBP from the perspective of their working conditions
Mozafari et al (2015)^1^	Iran	Case‐control	346	Truck	37	Determine the prevalence of MSDs and associated risk factors
Nazerian (2018)^71^	Turkey	Cross sectional	384	Crane	48	Assess the association of musculoskeletal discomfort with psychosocial and physiological factors
Okunribido, Magnusson and Pope (2006)^72^	UK	Cross‐sectional	64	Delivery van	47	Investigate exposures of posture demands, manual handling and WBV as well as prevalence and nature of LBP
Okunribido, Magnusson and Pope (2008)^73^	UK	Cross‐sectional	418	Various	47	Investigate prevalence and nature of LBP and determine relative importance of each risk factor associated
Okunribido, et al (2007)^74^	UK	Cross‐sectional	61	Bus	46	Investigate exposures of posture demands, manual handling and WBV as well as prevalence and nature of LBP
Porter and Gyi (2002)^75^	UK	Cross‐sectional	113	Car	39.3	Investigate the prevalence of MSK troubles and exposure to driving
Raanaas and Anderson (2008)^76^	Norway	Cross‐sectional	823	Taxi	43	Determine prevalence of MSK pain and identify occupational risk factors associated with neck, shoulder or low back pain
Robb and Mansfield (2007)^11^	UK	Cross‐sectional	192	Truck	45.8	Identify the prevalence of MSK problems and assess links between risk factors and back pain
Rufa'I et al (2015)^77^	Nigeria	Cross‐sectional	200	Car and minibus	42.4	Determine prevalence of LBP and identify associated risk factors and economic impact
Rugbeer, Neveling and Sandla (2016)^78^	South Africa	Cross‐sectional	89	Bus	45	Determine the prevalence of WRMDs in long‐distance bus drivers
Sang, Gyi and Haslam (2010)^79^	UK	Cross‐sectional	140	Car	38.2	Assess prevalence or MSKSs and associated risk factors among pharmaceutical sales representatives
Szeto and Lam (2007)^14^	Hong Kong	Cross‐sectional	481	Bus	47	Investigate prevalence and characteristics of occupational MSDs in male and female bus drivers
Sekkay et al (2018)^80^	Canada	Cross‐sectional	123	Truck	49	Document the prevalence of self‐reported MS pain in different body areas
Senthanar et al (2018)^81^	Canada	Cross‐sectional	107	Truck	45	Assess the prevalence of musculoskeletal pain and discomfort in Canadian truck drivers
Tamrin et al (2007)^38^	Malaysia	Cross‐sectional	760	Bus	43	Determine the prevalence of MSDs including LBP and revealing their physical and psychological risk factors
Tamrin et al (2014)^9^	Malaysia	Cross‐sectional	1180	Bus	NR	Determining the prevalence of MSDs and risk factors that may contribute to MSD problems
Wang et al (2017)^82^	China	Cross‐sectional	719	Taxi	40	Investigate the prevalence of LBP and associated work‐related risk factors among Chinese Taxi drivers
Yasobant et al (2015)^3^	India	Cross‐sectional	280	Bus	34	Assess the personal and ergonomic risk of developing work‐related MSDs among bus drivers

Abbreviations: N, participant sample number; NR, not reported.

### 3.3 Quality of reviewed articles

Of the 56 studies included in the study, seven were considered of low methodological quality with a very high risk of bias, 32 of medium methodological quality with a high risk of bias, and 17 studies were considered of high methodological quality with a low risk of bias. Selection bias was apparent in most of the cross‐sectional studies (Table 2, items 1 and 2) and many of them failed to provide an acceptable case definition (Table 2, item 6). Additionally, many prospective cohort studies presented the outcome of interest at the start of the study (Table 3A,B).

**TABLE 2:** Methodological quality scores of cross sectional studies

Author (year)	External validity				Internal validity						Quality
	1	2	3	4	5	6	7	8	9	10	
Abledu et al (2014a)^2^	N	N	N	N	Y	N	Y	Y	Y	Y	(5) Med
Abledu et al (2014b)^40^	N	N	N	N	Y	N	Y	Y	Y	Y	(5) Med
Akinpelu et al (2011)^41^	N	N	N	N	Y	N	Y	Y	Y	Y	(5) Med
Alperovitch‐Najenson et al (2010a)^15^	N	N	Y	N	Y	N	Y	Y	Y	Y	(6) Med
Alperovitch‐Najenson et al (2010b)^42^	N	N	Y	N	Y	N	Y	Y	Y	Y	(6) Med
Aminian et al (2016)^43^	N	N	N	Y	Y	N	Y	Y	Y	Y	(6) Med
Andrusaitis et al (2016)^45^	N	N	N	N	Y	Y	N	Y	Y	Y	(5) Med
Anjomshoae et al (2013)^46^	N	N	N	N	Y	N	Y	Y	Y	Y	(5) Med
Boshuizen et al (1990)^47^	N	N	N	N	Y	Y	N	Y	Y	Y	(5) Med
Boshuizen et al (1992)^48^	N	N	N	N	Y	Y	N	Y	Y	Y	(5) Med
Bovenzi et al (2006)^52^	Y	Y	Y	N	Y	Y	Y	Y	Y	Y	(9) High
Bovenzi and Betta (1994)^51^	N	N	N	N	Y	N	N	N	Y	Y	(3) Low
Bovenzi and Zadini (1992)^23^	N	N	N	Y	Y	N	Y	N	Y	Y	(5) Med
Burdorf, et al (1993)^54^	N	N	N	N	Y	Y	Y	Y	Y	Y	(6) Med
Burgel et al (2017)^55^	N	N	N	N	Y	N	Y	Y	Y	Y	(5) Med
Chen et al (2004)^16^	N	N	N	Y	Y	N	Y	Y	Y	Y	(6) Med
Chen et al (2005)^56^	N	N	N	Y	Y	N	Y	Y	Y	Y	(6) Med
Feng (2018)^57^	Y	Y	N	N	Y	Y	Y	Y	Y	Y	(8) High
Gangopadhyay and Dev (2012)^58^	N	N	N	N	Y	N	Y	Y	Y	Y	(5) Med
Geete et al (2013)^10^	N	N	N	N	Y	N	N	Y	N	Y	(3) Low
Greiner and Krause (2006)^59^	N	Y	Y	Y	Y	Y	N	Y	Y	Y	(8) High
Gyi and Porter (1998)^60^	N	N	Y	N	Y	N	Y	Y	Y	Y	(6) Med
Jadhav (2016)^61^	N	N	N	N	Y	N	N	Y	N	Y	(3) Low
Kaila‐Kangas et al (2011)^62^	Y	Y	N	Y	Y	Y	Y	Y	N	Y	(8) High
Kim et al (2016)^63^	N	Y	Y	N	Y	N	Y	N	N	Y	(5) Med
Krause et al (1997)^65^	N	N	N	Y	Y	Y	N	Y	N	Y	(5) Med
Laal et al (2017)^66^	N	N	Y	N	Y	Y	N	Y	Y	Y	(6) Med
Lalit et al (2015)^67^	N	N	N	N	Y	N	Y	Y	N	Y	(4) Low
Mansfield and Marshall (2001)^68^	N	N	N	N	Y	Y	Y	N	Y	Y	(5) Med
Miyamoto et al (2008)^69^	N	N	N	N	Y	N	Y	Y	Y	Y	(5) Med
Miyamoto et al (2000)^70^	N	N	N	N	Y	N	N	Y	Y	Y	(4) Low
Nazerian (2018)^71^	Y	Y	N	Y	Y	Y	Y	Y	Y	N	(8) High
Okunribido et al (2006)^72^	N	N	Y	N	Y	N	Y	N	Y	Y	(5) Med
Okunribido et al (2008)^73^	N	N	Y	N	Y	N	Y	N	Y	Y	(5) Med
Okunribido et al (2007)^74^	N	N	Y	N	Y	N	Y	N	Y	Y	(5) Med
Porter and Gyi (2002)^75^	Y	Y	Y	N	Y	N	Y	Y	Y	Y	(8) High
Raanaas and Anderson (2008)^76^	Y	Y	N	N	Y	N	Y	Y	Y	Y	(7) High
Robb and Mansfield (2007)^11^	N	Y	Y	N	Y	N	Y	Y	Y	Y	(7) High
Rufa'i et al (2015)^77^	N	N	N	N	Y	N	Y	Y	Y	N	(4) Low
Rugbeer et al (2016)^78^	N	N	N	N	Y	N	Y	Y	Y	Y	(5) Med
Sang et al (2010)^79^	N	N	Y	N	Y	N	Y	Y	Y	Y	(6) Med
Sekkay et al (2018)^80^	Y	N	N	Y	Y	Y	Y	Y	Y	Y	(8) High
Senthanar et al (2018)^81^	Y	Y	N	N	Y	Y	Y	N	Y	Y	(7) High
Szeto and Lam (2007)^14^	N	Y	N	N	Y	N	Y	Y	Y	Y	(6) Med
Tamrin et al (2007)^38^	N	Y	N	N	Y	N	Y	Y	Y	Y	(6) Med
Tamrin et al (2014)^9^	N	Y	Y	N	Y	N	Y	Y	Y	Y	(7) High
Wang et al (2017)^82^	N	Y	Y	N	Y	N	Y	Y	Y	Y	(7) High
Yasobant et al (2015)^3^	N	N	Y	N	Y	N	Y	Y	Y	Y	(6) Med

Note

High, high quality (low risk of bias); Low, low quality (high risk of bias); Med, medium quality (moderate risk of bias); N, no; Y, yes; 1—Was the study's target population a close representation of the national population in relation to relevant variables, age, sex, occupation? 2—Was the sampling frame a true or close representation of the target population? 3—Was some form of random selection used to select the sample, OR, was a census undertaken? 4—Was the likelihood of non‐response bias minimal? 5—Were data collected directly from the subjects (as opposed to a proxy)? 6—Was an acceptable case definition used in the study? 7—Was the study instrument that measured the parameter of interest (eg, prevalence of low back pain) shown to have reliability and validity (if necessary)? 8—Was the same mode of data collection used for all subjects? 9—Was the length of the shortest prevalence period for the parameter of interest appropriate? 10—Were the numerator(s) and denominator(s) for the parameter of interest appropriate? (Hoy et al 2012).

**TABLE 3A:** Methodological quality scores of case‐control studies

Author (year)	Overall Items									Quality
	1	2	3	4	5	6	7	8	9	
Anderson (1992)^44^	Y	Y	Y	Y	N	N	N	Y	Y	(6) Med
Magnusson et al (1996)^13^	N	N	N	N	N	N	N	Y	Y	(2) Low
Mozafari et al (2015)^1^	Y	N	Y	Y	Y	N	N	Y	N	(5) Med

Note

Low, low quality (high risk of bias); Med, medium quality (moderate risk of bias); N, no; Y, yes; 1—Case definition. 2—Representation of cases. 3—Selection of controls. 4—Definition of controls. 5—Study controls for important factor. 6—Study controls for an additional factor. 7—Ascertainment of exposure. 8—Same method of ascertainment used for both cases and controls. 9—Non‐response rate.^19^

**TABLE 3B:** Methodological quality scores of prospective cohort studies

Author (year)	Overall Items									Quality
	1	2	3	4	5	6	7	8	9	
Bovenzi (2009)^49^	Y	Y	Y	N	Y	Y	N	Y	Y	(7) High
Bovenzi (2010)^50^	Y	Y	Y	Y	Y	Y	N	Y	N	(7) High
Bovenzi (2015)^36^	Y	Y	Y	N	Y	Y	N	Y	Y	(7) High
Bovenzi et al (2015)^53^	Y	Y	Y	N	Y	Y	N	Y	Y	(7) High
Krause et al (1998)^64^	Y	Y	Y	N	Y	Y	N	Y	Y	(7) High

Note

High, high quality (low risk of bias); Low, low quality (high risk of bias); Med, medium quality (moderate risk of bias); N, no; Y, yes; 1—Representativeness of the exposed cohort. 2—Selection of the non‐exposed cohort. 3—Ascertainment of exposure. 4—Demonstration that outcome of interest was not present at the start of study. 5—Study controls for important factor. 6—Study controls for an additional factor. 7—Assessment of outcome. 8—Was follow‐up long enough for outcomes to occur? 9—Adequacy of follow‐up cohorts.

### 3.4 Prevalence of musculoskeletal pain among professional drivers

All studies included investigated prevalence rates of musculoskeletal pain (MSP) among professional drivers (Table 4). Of these 56 studies, 18 studies (N = 6588) reported total prevalence rates of MSP ranging between 43.1% and 93%, with a mean of 73%. The low back region was the most frequently reported body region, with 43 studies (N = 9998) reporting a prevalence rate of LBP between 17% and 82.9%, with a meta‐prevalence rate of 53%. Twenty‐six studies (N = 3480) reported prevalence of neck pain between 7.1% and 78.8% with a meta‐prevalence rate of 42.4%. Shoulder pain was reported between 6.3% and 79.4% in 19 studies (N = 2751) with a meta‐prevalence of 39.2%. Fourteen studies (N = 1299) reported prevalence of upper back pain between 2.6% and 60.3% with an estimated meta‐prevalence rate of 25.5% and 16 studies (N = 1460) reported knee pain prevalence between 5.6% and 36% with an estimated meta‐prevalence of 21.8%. Hip/thigh pain was reported with a prevalence between 2.7% and 22.2% with a meta‐prevalence of 19.5% in eight studies (N = 655). Wrist pain prevalence ranged between 1.3% and 31% in nine studies (N = 239), reporting an estimated meta‐prevalence of 11.5%. The other body regions affected were ankle (N = 266) and elbow (N = 313) and these studies reported an estimated meta‐prevalence of 15.1% and 7.9%, respectively.

**TABLE 4:** Prevalence rates of musculoskeletal disorders among professional drivers

Author (year)	Vehicle types	Prevalence duration	Overall study quality	Total MSD prevalence	Results of prevalence rates
Abledu, Offei and Abledu (2014a)^2^	Minibus	12‐mo	(5) Med	78.40%	Pain in low back 58.8%, neck 25%, upper back 22.3%, shoulder 18.2%, knee 14.9%, ankle 9.5%, wrist 7.4%, elbow 4.7%, hip/thigh 2.7%
Abledu, Offei and Abledu (2014b)^40^	Taxi	12 mo	(5) Med	70.50%	Pain in low back 34.3%, upper back 16.7%, neck 15.2%
Akinpelu, et al (2011)^41^	Various	12‐mo	(5) Med	89.30%	Shoulder 11%, knee 10%, hip/thigh 2.9%, elbow 4.8%, ankle/feet 2.4%, wrist/hand 1.9% Pain in low back 64.8%, shoulder 30.8%, knee 27.0%, neck
Alperovitch‐Najenson et al (2010a, 2010b)^15^, ^42^	Bus	12‐mo	(6) Med	NR	17.0%, upper back 2.6%, Pain in low back 45.4%, neck 21.2%, shoulder 14.7%
Aminian et al (2016)^43^	Truck andTaxi	12‐mo	(6) Med	NR	Upper back 8.3%, elbow 3.0%, wrist 3.0%. pain in neck 11.5%, upper back 9.6%, low back 19.5%, knees 9.3% Taxi drivers: pain in neck 2.7%, upper back 8.2%, low back 14.4%, knees 1.9%
Anderson (1992)^44^	Bus	Point prevalence	(6) Med	80.50%	Pain in back 66.4%, neck 50.8%. Spine pain 80.5%
Andrusaitis et al (2006)^45^	Truck	During work	(5) Med	NR	59% LBP
Anjomshoae et al (2013)^46^	Bus	12‐mo	(5) Med	NR	Pain in shoulder 79.4%, neck 66.4%. upper back 60.3%
Boshuizen et al (1990)^47^	Tractor	12‐mo	(5) Med	NR	Back 22.9%, ankle/feet 12.2%, wrist/hands 11.5%, knee, 9.9%, thigh/hips 4.6%, elbow 3.1%. Pain in back 38.4%, low back 31.3%
Boshuizen et al (1992)^48^	Truck	12‐mo	(5) Med	NR	51% LBP
Bovenzi (2009, 2010, 2015)^36^, ^49^, ^50^	Various	12‐mo	(7) High	NR	LBP ‐ 64.4% prevalence, 36.3% incidence, Neck pain ‐ 31.9% incidence, 78.8% prevalence Shoulder pain ‐ 21.4% incidence, 65.3% prevalence
Bovenzi and Betta (1994)^51^	Tractor	Lifetime	(3) Low	86.10%	LBP ‐ 81.3% lifetime; 71.9% 12 mo; 39.2% 1 mo
Bovenzi et al (2006)^52^	Various	12‐mo	(9) High	NR	LBP in vehicle populations: Bus 71.4%; garbage 61.3%; paper mills 62.8%; dockyards 53.3%; marble labs 55.4%
Bovenzi et al (2015)^53^	Various	12‐mo	(7) High	NR	Marble quarries 58.2% 23.1% sciatic pain
Bovenzi and Zadini (1992)^23^	Bus	12‐mo	(5) Med	NR	82.9% LBP
Burdorf et al (1993)^54^	Straddle‐carrier	12‐mo	(6) Med	NR	44% LBP
Burgel et al (2017)^55^	Taxi	12‐mo	(5) Med	NR	63% LBP
Chen et al (2004, 2005)^16^, ^56^	Taxi	12‐mo	(6) Med	NR	51% LBP, 19% knee pain
Feng (2018)^57^	Taxi	12‐mo	(8) High	NR	LBP 58%, NP 56.8%,SP 43.2%, H&TP 29.6%, A&FP 21%, Upper back pain 11%, EP 4.9%, KP 4.9%, W&HP 2.5%
Gangopadhyay et al (2012)^58^	Bus	12‐mo	(5) Med	NR	73% LBP
Geete et al (2013)^10^	Bus	Unclear	(3) Low	80%	Pain in low back 70%, neck 55%, shoulder 47.5%,
Greiner and Krause (2006)^59^	Transit	12‐mo	(8) High	49%	Pain in low back 32.3%, neck 18.5%, upper extremity 27.3%, lower extremity 30.8%
Gyi and Porter (1998)^60^	Police car	Lifetime	(6) Med	65%	29% LBP
Jadhav (2016)^61^	Bus	10‐y	(3) Low	NR	70.8% LBP
Nazerian (2018)^71^	Crane	12‐mo	(8) High	NR	LBP 57%, NP 55%,SP 87%, Buttock pain 32%, A&FP 26%, Upper back pain 33%, EP 21%, KP 45%, W&HP 34%
Kaila‐Kangas et al (2011)^62^	Various	Lifetime	(8) High	NR	4.0% Chronic LBP
Kim et al (2016)^63^	Truck	Unclear	(5) Med	NR	LBP 72.5%. shoulder pain 55.1%, neck pain 50.7%
Krause et al (1998)^64^	Transit	5‐y	(7) High	77.7%	Spinal injury 76.9%
Krause et al (1997)^65^	Transit	Point prevalence	(5) Med	NR	Back and neck pain 14%
Laal et al (2017)^66^	Bus	Point prevalence	(6) Med	NR	Severe LBP 33.3%, upper back pain 18.3%, knee pain 15%.
Lalit, Soni and Garg (2015)^67^	Bus	NR	(4) Low	53%	Pain in low back 30.3%, neck 17.3%, knee 14.7%, shoulder6.3%, ankle and feet 5.7%, upper back 4%, hip and thigh4%, elbow 1.3%, wrist and hand 1.3%
Magnusson et al (1996)^13^	Bus and Truck	Point prevalence	(2) Low	NR	Truck drivers pain: Low back 56%, neck 36%, shoulder 37% Bus drivers pain: Low back 60%, neck 45%, shoulder 36%
Mansfield et al (2001)^68^	Racing Car	12‐mo	(5) Med	NR	70% LBP, 54% cervical spine pain
Miyamoto et al (2008)^69^	Taxi	1 wk	(5) Med	NR	20.5% LBP
Miyamoto et al (2000)^70^	Truck	1 mo	(4) Low	NR	50.3% LBP
Mozafari et al (2015)^1^	Truck	12‐mo	(5) Med	78.6%	24.3% LBP, neck pain = 27.2%
Okunribido et al (2006)^72^	Delivery Van	12‐mo	(5) Med	NR	50% LBP
Okunribido et al (2008)^73^	Various vehicles	12‐mo	(5) Med	NR	55.7% LBP
Okunribido et al (2007)^74^	Bus	12‐mo	(5) Med	NR	59% LBP
Porter and Gyi (2002)^75^	Car	12‐mo	(8) High	NR	61% LBP
Raanaas et al (2008)^76^	Taxi	12‐mo	(7) High	NR	Pain in low back 59.5%, shoulder 52.4%, neck 57.8%.
Robb et al (2007)^11^	Truck	12‐mo	(7) High	81%	Pain in low back 60%, neck 34%, shoulder 39%, knees 36%, wrist/hands 20%, hips 15%, upper back 14%, ankles/feet13%, elbows 9%.
Rufa'I et al (2015)^77^	Car and mini bus	12‐mo	(4) Low	NR	73.5% LBP
Rugbeer et al (2016)^78^	Bus	Unclear	(5) Med	67%	Pain in upper back 44%, lower back 42%, neck 42%, shoulder 37%, wrist/hand 31%
Sang et al (2010)^79^	Car	12‐mo	(6) Med	84%	Pain in low back 57%, neck 46% and shoulder 45%
Senthanar et al (2018)^81^	Truck	12‐mo	(7) High	57%	Shoulder 54%, wrist/hands 44%, upper back 39%, lower back 80%, legs/feet 41%
Sekkay et al (2018)^80^	Truck	12‐mo	(8) High	43.1%	Neck 14.6%, shoulders 20.3%, upper back 6.5%, arms 8.1%, elbows 5.7%, lower back 21.1%, forearm/wrist/hand 12.2%, hip/thighs 8.9%, ankles/feet 5.7%
Szeto and Lam (2007)^14^	Bus	12‐mo	(6) Med	93%	Discomfort in low back 61%, neck 52%, shoulder 48%, thigh and knee 36%
Tamrin et al (2007)^38^	Bus	12‐mo	(6) Med	NR	Pain in low back 60.4%, neck 51.6%, shoulder 35.4%, upper back 40.7%, knee 29.3%
Tamrin et al (2014)^9^	Bus	12‐mo	(7) High	81.8%	Pain in low back 58.5%, neck 51.7%, shoulder 36.1%, elbow 10.2%, arm 17.5%, upper back 39%, hip and thigh 19.9%, knee 27.5%, leg 28.9%
Wang et al (2017)^82^	Taxi	12‐mo	(7) High	NR	54% LBP
Yasobant et al (2015)^3^	Bus	12‐mo	(6) Med	NR	Pain in neck 26%, back 24%, upper limb 20%

Abbreviation: LBP, low back pain; NR, not reported.

**FIGURE 2:**
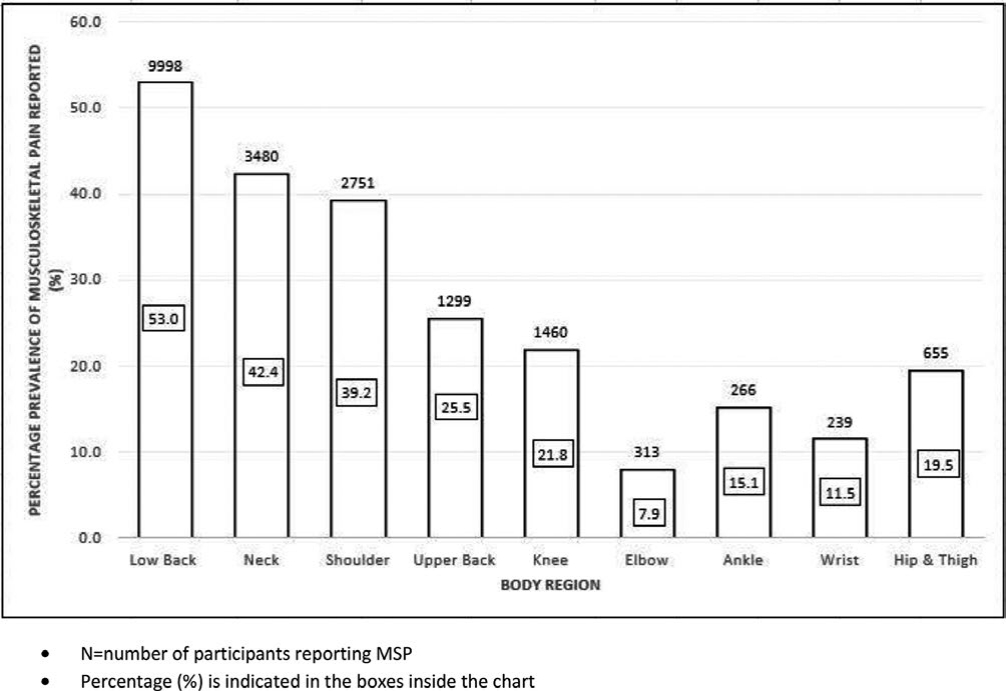
Prevalence of musculoskeletal pain (MSP) reported among professional drivers across specific body regions

**FIGURE 3:**
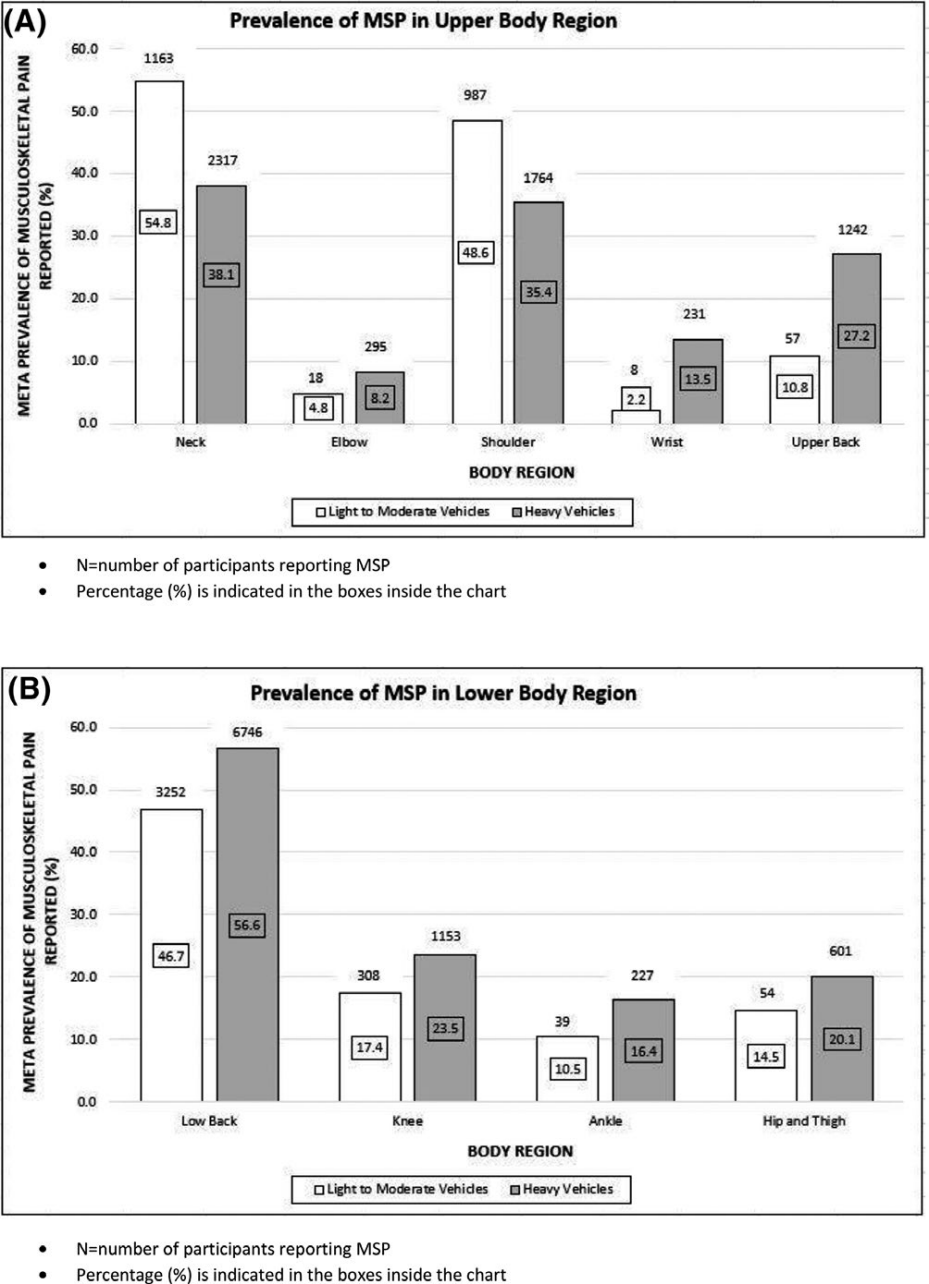
Subgroup analysis: Prevalence of musculoskeletal pain (MSP) in (A) upper body region and (B) lower body region reported among professional drivers driving light‐moderate and heavy vehicles

A summary of the overall breakdown of meta‐prevalence rates for specific body regions among professional drivers is presented in Figure 2. The results of the sub analysis of MSPs reported about professional drivers who drive light‐moderate vehicles and heavy vehicles are presented in Figure 3A,B.

## 4 DISCUSSION

### 4.1 Prevalence of MSP among professional drivers

The purpose of this review was to investigate the prevalence of MSP among professional drivers. An international expert group on WRMSDs initiated the current systematic review to identify the magnitude and possible variability of MSP among professional drivers globally. In the absence of comprehensive evidence on MSP among professional drivers, it is difficult to coordinate and provide appropriate services for the management of MSP in this group. The findings of this review provides comprehensive, comparable data on the prevalence of MSP among professional drivers.^8^ These findings can support policies and practices of policy makers and occupational health authorities, such as the HSE in the United Kingdom, and similar bodies in other countries to understand and address MSP among professional drivers, leading to improvement in the health of this working population.

The authors of this review consider this to be the first systematic review of epidemiological literature investigating the prevalence of MSP among this group of professionals. Heterogeneity was noted in the scope of reported prevalence, ranging from point prevalence to lifetime prevalence (Table 4). The most widely applied approach for estimating prevalence among professional drivers was 12‐month prevalence ranging from 43.1% to 93%. Within self‐reported epidemiological studies, longer prevalence periods increase the likelihood of participants being unreliable in recalling experiences of MSP.^18^ It is suggested that in order to reduce risk of bias, reporting 12‐month prevalence is an adequate period and can help establish standard reporting procedures, making the data comparable globally with a greater precision of prevalence. Many cross‐sectional and case‐control studies included in this review reported that professional drivers had higher MSP prevalence than non‐professional driver controls.^1^, ^22^, ^23^ The 12‐month prevalence of MSP in professional drivers ranged from 43.1% to 93%. This may indicate that professional drivers are at a particular risk of developing MSP compared with other occupational groups. While the prevalence of MSP is commonly reported among professional drivers, the magnitude of disability caused by MSP is unclear. Further studies are needed to understand the risk factors associated with MSP among drivers, the impact of MSP on the mental health, job satisfaction and sickness absenteeism of drivers. Also, differences in how the presence of MSP or musculoskeletal disorders is defined and how work‐relatedness is determined among observational studies is an important topic that needs further investigation. It is important to note that such a review had not been conducted so far in the field of musculoskeletal disorders related to work.

### 4.2 Prevalence of LBP

Previous research indicates that low back, neck and shoulder regions are the most commonly reported body regions affected by MSP.^24^ The present review found that the low back was the most commonly affected region, followed by neck and shoulder. The review findings suggest that professional drivers have a higher prevalence rate for LBP (53%) when compared with other occupations such as manual material handling workers (25%) and physiotherapists (50%).^25^, ^26^ When compared to the prevalence rates (38%) reported globally for LBP,^27^ the higher rate observed among professional drivers indicates the significance of the problem for this occupational group. Findings in this review are complemented by a recent smaller review reporting a high prevalence of LBP and neck pain among bus drivers.^28^ With a much larger pooled sample (N = 18 882), the current review reports a meta‐prevalence of LBP (53%), consistent with the high prevalence of LBP reported in other recent professional driving studies^29^, ^30^, ^31^ at 61.7%, 49%, and 54% respectively.

Another systematic review of the global prevalence of LBP reported a mean 12‐month prevalence of 38.9%.^32^ This suggests the increased risk of occupational groups, such as professional drivers, developing low back symptoms and disorders. The findings from the current review subgroup analysis (Figure 3A,B) showed a similar trend in the prevalence of LBP in drivers of both low‐moderate vehicles and heavy vehicles.

The high prevalence of LBP is considered to place great financial burden on, and possibly contribute to early retirement, among such individuals.^32^, ^33^ The findings on LBP from this review would support action by the relevant transport‐occupational health sectors toward the prevention and management of LBP among such drivers. It is suggested that the current review findings might prompt practitioners and policy makers across different countries to understand and act appropriately to improve overall health care and wellbeing of professional drivers.

### 4.3 Prevalence of MSP in the upper body region

The 12‐month reported prevalence of neck pain ranged between 7.1% and 78.8%. A previous synthesis of 249 papers reported a 12‐month prevalence of neck pain similarly ranging between 12.1% and 71.5% in the general population,^34^ with most estimates ranging between 30% and 50%. Our estimated meta‐prevalence of 42.4% is comparable to a 12‐month global mean prevalence of 37.2%.^35^ This seems to indicate that professional drivers are at a higher risk of developing neck pain than the general public. The large variations of prevalence estimates in the general population have been credited to differences in the demographic and socio‐economic status of the surveyed populations, methods of case definition, and ascertainment and the criteria for inclusion/exclusion in various studies.^34^, ^36^ Similarly, these variables could also contribute to the large variations of prevalence estimates presented in this review.

In the present review, the meta‐prevalence of MSP in the neck, shoulder, and upper back region was 42.4%, 39.2%, and 25.5% respectively. The prevalence of shoulder pain among drivers is higher than the global 12‐month prevalence of shoulder pain in the general public (36.7%).^37^ Although three included studies reported higher prevalence of MSP in the upper back than the shoulder region,^2^, ^9^, ^38^ the meta‐prevalence of MSP as indicated in Figure 2 suggests that the shoulder region is more affected than the upper back. It is noteworthy that the subgroup analysis from Figure 3A,B indicated prevalence of MSP in the neck and shoulder regions as noticeably higher among drivers in the light‐moderate vehicles sector compared to drivers using heavy vehicles. Involvement of neck, shoulder, and upper back pain has been well documented among professional bus driver^15^ and significant prevalence of this body region reported among professional drivers in general, demonstrates a clinical need to investigate the biomechanics of the upper quadrant region among this group. Currently, there is a paucity of evidence available for practitioners to understand the pathogenesis of upper quadrant musculoskeletal problems among professional drivers. It is suggested that future research on the kinetics and kinematic parameters of scapula kinesis and their association with musculoskeletal symptoms is warranted. Furthermore, available scientific evidence on drivers who experience neck pain, also shows a higher prevalence of upper back and shoulder pain compared to drivers without neck pain,^15^ and this warrants investigation of the upper quadrant motor control mechanisms among professional drivers.

### 4.4 Prevalence of MSP in the lower extremity region

This review reported an estimated meta‐prevalence of 21.8% knee pain and 19.5% hip pain, demonstrating that MSPs are common in lower extremity regions among professional drivers. The prevalence of MSPs in the lower extremity regions were generally higher among heavy vehicle drivers compared to those driving low‐moderate vehicles. As shown in Figure 3B, the ankle was the least affected region in the lower extremity with a meta‐prevalence rate of 10.5% and 16.4% among drivers driving low‐moderate and heavy vehicles, respectively. Some of the MSP experienced in the upper and lower extremities might possibly be referred pain from the spine, or have local origin due to mechanical loading of these joints associated with postures and repetitive movements involved in professional driving.^14^ Generally, the studies included in this review did not clarify the spinal origin of pain in the extremities and hence, no conclusions can be drawn for the spinal contribution of MSP to pain in extremities related to professional driving.

### 4.5 Implications for practice

The findings of this review provide scientific evidence internationally to stake holders such as policy makers, insurance providers, occupational health authorities, researchers, and clinicians on the magnitude of MSP among professional drivers. Aside from providing scientific evidence on MSP to those concerned, the review findings raise several other key points for consideration. First, the availability of prevalence data alone may not be helpful in planning health interventions or policies, but the review findings point to a need to investigate risk factors that contribute to MSP among professional drivers so that adequate interventions could be designed to address this global issue. Further investigation of the multifaceted and complex risk factors and contributors to MSP is needed before work‐place interventions can be attempted. Therefore, a systematic review to investigate risk factors and their causal relationship with MSP is urgently needed to assist policy makers in identifying and handling risk factors appropriately. Second, the biopsychosocial model needs to be considered when investigating management strategies for MSP in drivers.^39^ Drivers' perceptions and experiences of MSP, including pain and other symptoms have not been studied and therefore need to be explored further to fully understand the phenomenon. Additionally, drivers' perceptions of health and wellbeing, work/life balance, and mental attitude might all contribute to MSP outcomes and further study could assist policy makers and health authorities to understand the needs and expectations of drivers with MSP. The professional drivers themselves should be involved in framing management strategies to combat MSP.

### 4.6 Study limitations

The review highlighted some limitations. The use of the term “work related musculoskeletal disorders” was not consistent between the studies included. Often interchangeable terms such as: work‐related musculoskeletal symptoms, musculoskeletal complaints, musculoskeletal problems, MSP, and musculoskeletal discomfort were used across studies. Nevertheless, the studies reported widely the presence of pain as one of the commonest presentations among participants. Thus, MSP was considered as an umbrella term and a main outcome of interest for the current review. However, it is possible, that some studies included cases of non‐specific MSP that were not definitively linked to work. The absence of standardized methods across the studies and lack of general consensus to distinguish non‐occupational MSP indicates the challenge in estimating prevalence of MSP among drivers.

It is possible that other tasks, in addition to driving, (eg, loading and unloading trucks) may have occurred in the different groups of professional drivers. However, neither the current review nor the studies which reported MSP among drivers accounted for any procedures to address this confounding factor. Because of the limitations discussed above, the reviewers urge some caution in interpreting the prevalence of MSP among professional drivers.

## 5 CONCLUSION

The findings from the review provide evidence on the prevalence and severity of MSP among professional drivers. A wide range of prevalence rates of MSP affecting different body regions have been reported, with the highest prevalence found in the low back region, followed by the neck, shoulder and upper back regions. The review findings suggest that further investigation into the multiplicity of risk factors for MSP is necessary so that policy makers, health professionals, drivers themselves, and other stake holders can work together toward combatting MSP among this population.

## DISCLOSURE

*Approval of the research protocol*: N/A. *Informed consent*: N/A. *Registry and the registration no. of the study/trial*: N/A. *Animal studies*: N/A. *Conflict of interest*: Authors declare no conflict of interest for this article.

## AUTHOR CONTRIBUTIONS

LJ and MS contributed to the conception and design of the work; LJ, MS, AP, PS, and UP contributed to the acquisition, analysis, and interpretation of data for the work; LJ, MS, RK, AP, PS, and UP contributed to the drafting of the work or revising it critically for important intellectual content; LJ, MS, RK, AP, PS, and UP contributed to the final approval of the version to be published; LJ, MS, RK, AP, PS, and UP are accountable for all aspects of the work in ensuring that questions related to the accuracy or integrity of any part of the work are appropriately investigated and resolved.

## Supporting information

Supplementary Material
